# Gendered cultural context as a structural determinant of sexual and reproductive health

**DOI:** 10.1186/s12978-025-02188-7

**Published:** 2025-12-02

**Authors:** Zeinab Khadr, Hoda Rashad, Sherine Shawky

**Affiliations:** 1 Faculty of Economics and Political Science, Cairo University, Cairo, Egypt; 2 The Social Research Center, The American University in Cairo, Cairo, Egypt

**Keywords:** Gender equity, Cultural norms, Reproductive health risks, Structural determinants

## Abstract

**Background:**

The impact of cultural norms on women’s health, particularly their reproductive health, has long been acknowledged. However, due to extensive data requirements, creating an index to capture these cultural norms at the national level has proved challenging, making it difficult to calculate these indices for small communities within a nation. This paper proposes a new contextual index, which uses data from regularly fielded large-scale surveys, to evaluate the restrictive cultural rules that limit women’s health and well-being opportunities within the context of their small communities.

**Methods:**

The GCCI is a compositional index that combines the prevalence of five culturally restrictive women’s attributes at the community level using factor analysis. Egypt was adopted as a case study using data from the Egyptian Family Health Survey 2021. The Wagstaff concentration index assessed the GCCI’s validity in reflecting the inequalities in reproductive health risks. Multilevel logistic analysis was used to determine the index’s ability to explain the community-level variability in multiparity (having 5 + children).

**Results:**

The five indicators showed high internal consistency, with a Cronbach’s alpha of 0.72. The factor combining the five indicators accounted for 72.7% of the total variances. The index was effective in capturing the inequality in reproductive health risks among the 47 communities in Egypt, with a positive Wagstaff concentration index that exceeded 10% for the move from the least to the most restrictive communities in Egypt. The multilevel analysis showed that the community level captured 11% of the variability in multiparity, of which the GCCI index explained 51%. This suggests that the GCCI index can account for a significant portion of the community-specific factors that contribute to reproductive health disparities.

**Conclusion:**

The GCCI index, with its simple structure and reliance on regularly available large-scale data, offers strong evidence of the significant effect of the gendered cultural context in explaining inequality in women’s reproductive health risks. It has successfully pinpointed communities that limit women’s health and well-being opportunities. Adopting a similar approach to capture the cultural context could provide substantial support for the development of culturally sensitive and tailored interventions that address the cumulative effects of restrictive cultural norms across different communities.

## Abstract (plain language summary)

The impact of cultural norms on women’s health, particularly their reproductive health, has long been acknowledged. However, due to extensive data requirements, creating an index to assess these restrictive cultural norms at the national level has proved challenging. This has made it difficult to calculate these indexes for small communities within a nation. This paper proposes a new contextual index to evaluate the restrictive cultural rules that limit women’s health and well-being opportunities within the context of their small communities. The new CGGI index is a composite index at the community level, reflecting the collective attributes of the community regarding women’s position in society and the household. Data for these attributes are regularly available in large-scale data sets conducted in many developing countries, such as the Demographic and Health Surveys. These attributes pertain to early marriage, approval of wife-beating, lack of decision-making power, exposure to violence, and low educational attainment. The index was applied to the 2021 Egyptian Family Health Survey data and successfully identified inequality in reproductive health risks across the index range. It also explained more than half of the community-based variability in having five or more children. These findings show that adopting similar indexes that capture the social and cultural norms in communities can help identify vulnerable communities where women are more exposed to reproductive health risks due to their cultural context, enabling the proper tailoring of interventions and service provision.

## Background

Gender equity in health has long been a focus for researchers [1–9]. However, while gender is acknowledged as a cultural concept, many studies on health inequalities focus on biological sex and use socioeconomic status as a stand-in for gender [10]. This emphasis on biological sex focuses mainly on individual-level attributes and limits the investigations to differences between men and women. Amid the current shift in the study of health disparities, which calls for tracing these disparities to structural root causes [11–13], this focus on individual-level attributes overlooks the power dynamics and the influence of cultural gender norms on access to resources and health opportunities for women and men [14]. More importantly, it disregards differences in access to these resources and opportunities within the same gender. There is a need for measures that capture these differences, allowing for varying classifications between and within genders and guiding more effective action to address health inequities.

Recently, researchers have introduced various concepts that highlight contextual factors contributing to health inequalities. One such concept is “constrained choices,” which encompasses contextual measures reflecting the impact of family, community, and social policies on gender-based health differences [15]. Another concept, “social location,” as defined by Lynam and Cowley [16], describes a process of “othering” that limits opportunities and restricts choices in the context of marginalization and health. This is represented through categorizations related to access to resources and social connections that contribute to social acceptance. In a study on racial health differences, Cogburn [17] presented the concepts of “stereotype threat” and “situational threat.” “Stereotype threat” refers to situations where individuals perceive others through stereotypes. In contrast, “situational threat” relates to prevailing negative attitudes toward certain groups, leading to societal conditions, cultural norms, and institutional practices that limit opportunities and resources for these groups. Both threats significantly restrict life opportunities and well-being for individuals [18]. The literature strongly recommends re-evaluating the concept of a culture of power and discrimination, aiming to encompass the array of cultural constructs that impact the health of specific social groups.

The study of gender, in particular, has led to the development of various approaches, with significant contributions from the sociological and social psychology literature [19]. Two influential approaches are “gender trouble” and the interactive model of gender-related behavior [20]. These approaches argue that gender stereotypes are shaped by specific social norms, defining acceptable gender roles and behaviors based on existing rules, normative expectations, and structural factors. Deviation from these norms often leads to social sanctions and contributes to differences in power and status based on gender. Similarly, researchers have proposed the concept of a gender system, which views gender as a structural force shaping social practices differently for men and women, leading to inequality within and between genders [21]. They suggest that the gender system operates at multiple levels – macro, relational, and individual – influencing choices and outcomes, including health, for men and women. The macro level sets cultural guidelines, defining the attributes of men and women and their roles, while the relational level outlines the social settings in which these roles are carried out, with variations across different communities due to cultural alignment.

Although many researchers have acknowledged the effect of the cultural rules within the gender system on achieving full potential outcomes, most of the efforts to capture these rules have focused on comparing countries [10, 22]. The United Nations Development Programme’s (UNDP) Gender Inequality Index is one example of these efforts [23]. With gender-specific indicators assessing the three dimensions of health, empowerment, and the labor market, the index captures the inequality in gender-specific outcomes. Still, it does not examine the underlying cultural beliefs that contribute to gender-differentiated outcomes. Additionally, the macro nature of the indicators selected, particularly those in the empowerment category, limits the applicability of this index to the national level, assessing inequalities by comparing different countries or regions. Another example is the OECD Development Centre’s Social Institutions and Gender Index (SIGI), proposed in 2009 to evaluate the gender-based discrimination exerted by formal and informal social institutions that undermine women’s opportunities to achieve their potential [24]. Covering four dimensions (discrimination in the family, restricted physical activity, limited access to productive and financial resources, and restricted civil liberties), 16 indicators, and 25 variables, the index requires a comprehensive information base that hinders its calculation for 39 countries and complicates its calculation for small communities within countries. Another example is the Gender Social Norms Index (GSNI), which UNDP launched in its 2019 Human Development Report. It aims to capture the social beliefs, biases, and prejudices that shape women’s roles across four dimensions: political, educational, economic, and physical integrity. The index is based on individuals’ ratings of seven statements, reflecting its four dimensions, as part of the World Values Survey, which explores changes in cultural values over time across different countries. Despite its relevance in addressing social norms, the index is based on a perception-based assessment of these norms, rather than an action-based evaluation. The approach does not permit policy interventions to address these norms [25]. Country-based measures and data requirements like those deployed by the indices outlined above have limited applicability in small communities. They cannot be used as a structural determinant of health disparities.

The current study proposes a straightforward approach to developing a new index that captures the macro and relational levels of the gender systems in small communities within a country. The Gendered Cultural Context Index (GCCI) is a simple composite index that captures the cultural beliefs that shape gender roles in these communities. These gender roles are a significant part of the context underlying women’s health and well-being. As a context-related index, the GCCI indirectly affects reproductive health by affecting individual women’s opportunities and access to resources. These limited opportunities and access to resources are reflected in the availability of limited facilities serving women and reduced opportunities that hinder their progress and development, ultimately affecting their individual characteristics. The main advantage of the GCCI is its simplicity; it can be developed using simple variables commonly available in large-scale population-based datasets, such as the multi-round Demographic and Health Surveys and family and population health surveys. The index focuses on the cumulative impact of cultural elements that undermine women’s opportunities to achieve their full potential contribution to society and development efforts.

## Gendered Cultural Context Index

The GCCI is a simple composite index that captures cultural rules at the level of small communities. These cultural rules are believed to restrict women’s opportunities to achieve their potential health outcomes. The index structure is based on social norm theories, which maintain that descriptive and injunctive norms shape people’s beliefs [26–28]. Descriptive norms focus on the commonly practiced behaviors within a social group, while injunctive norms are primarily concerned with the approval or disapproval of specific behaviors within social groups. This section presents the methodology used to develop the GCCI, using data from the Egyptian Family Health Survey 2021.

### Choice of indicators

The structure of the GCCI is based on combining the prevalence of five women-specific attributes at the community level. These attributes pertain to child marriage, exposure to violence, approval of wife-beating, lack of decision-making power, and low educational attainment. The literature provides overwhelming evidence that these attributes are strongly interlinked and can be traced to prevailing social norms in the communities promoting males’ dominating and controlling behavior over women and ensuring women’s subservience, which in turn disempowers women and denies them their basic human rights. Low educational attainment, approval of wife-beating, and lack of decision-making power undermine women’s agency and voice and pave the way to many harmful practices against them, including child marriage and violence against women [29–34]. All attributes have been linked to substantial long- and short-term negative consequences for women’s health and welfare, including significant physical and psychological problems, as well as many issues in their social and family context [35].

## Methods

The prevalences of the five attributes were aggregated at the community level. Their internal consistency was then tested using Cronbach’s alpha. This was followed by a one-factor exploratory factor analysis using the principal factor extraction method, with orthogonal rotation applied to the aggregated prevalences of the attributes.

A descriptive analysis illustrates the relationship between the GCCI and women’s reproductive health. For this purpose, the index was categorized into several homogeneous clusters using the K-means cluster analysis, producing an ordinal score for the index, referred to as the Gendered Cultural Context Score (GCCS). The optimal number of clusters was identified using the Calinski-Harabasz pseudo-F index. The Calinski-Harabasz pseudo-F index is a commonly used measure in cluster analysis to assess the distinct partitioning of the data, with larger values indicating a better partition [35–37]. For a dataset of n points, partitioned into k clusters:

$$\:CH\left(k\right)=\frac{{B}_{k}/(k-1)}{{W}_{k}\:(n-k)}\:\:\:\:\:$$

Where.

n = number of observations.

k = number of clusters.

B_k_​ = between-cluster dispersion (sum of squared distances between each cluster mean and the overall mean, weighted by cluster size).

W_k_​ = within-cluster dispersion (sum of squared distances between each point and its mean).

The association of the GCCS with several reproductive health dimensions was assessed to test its ability to reflect inequality among different types of communities. The Wagstaff concentration index for health inequality was also calculated to determine the level of inequality. The Wagstaff concentration index (CI) is an adaptation of the Gini coefficient to assess the relative distribution of health across an ordinal measure of social groups, ranging from the most vulnerable to the least vulnerable [38, 39]. It ranges between − 1 and + 1, with zero indicating the absence of inequality, a negative sign indicating high concentration in the most vulnerable social groups, and a positive sign indicating low concentration in these groups. The index works well for continuous health variables, but needs to be adjusted for binary variables by dividing its value by |1-$\:\mu\:$|to ensure its boundedness [40, 41].

$$\:CI=\frac{2}{\mu\:}\:COV({y}_{i},{R}_{i})$$

Where:

y_i_​ = health variable for individual i.

µ = mean of health variable y.

R_i_ = fractional rank of individual i in the ordinal measure of the social group distribution (most vulnerable = 0, least vulnerable = 1).

Additionally, as a contextual measure at the community level, the index’s ability to explain part of the variation in women’s reproductive health beyond individual attributes was tested using multilevel logistic regression. Controlling for individual attributes at the first level and using the communities at the second level, the GCCS was added to test its contribution in explaining some community-level variation. A multilevel logistic regression was applied to a critical dichotomous variable in sexual and reproductive health (SRH): multiparity, defined as 1 = having five or more children and 0 = otherwise. The regression controlled for five individual-level attributes: women’s age (centered at the grand mean), educational attainment (0 = less than secondary, 1 = secondary, and 2 = university and above), marrying at age < 18, experience of pregnancy loss or child death, and household wealth quintiles.

## Gendered Cultural Context Index for Egypt

To illustrate the construction of the GCCI and assess its feasibility for capturing inequalities in women’s reproductive health, the GCCI was developed for Egypt using data from the Egypt Family Health Survey 2021. The Egyptian Family Health Survey is a nationally representative survey that follows a similar data structure to that implemented in the Demographic and Health Surveys [42]. The objective of the Egyptian Family Health Survey 2021 was to provide population and reproductive health estimates at the national level. It also provides estimates at the level of the six regions of Egypt – urban governorates, urban Lower Egypt, rural Lower Egypt, urban Upper Egypt, rural Upper Egypt, and frontier (border) governorates – as well as at the level of 26 governorates (excluding one of the 27 total, North Sinai governorate).

The Egyptian Family Health Survey 2021 utilized the same design as the previous Demographic and Health Surveys. The design is based on a multi-stage stratified cluster sample. It covered the 26 governorates and included an urban/rural breakdown, providing estimates of the health indicators at the level of each governorate’s urban and rural areas. The final design included 47 strata since four governorates – Cairo, Alexandria, Port Said, and Suez – are urban governorates, and the rural areas in the Red Sea governorate were minimal and not included in the sample frame. A subsample representing half of the households in each enumeration area was selected to respond to the domestic violence module.

The sample size was not proportionally allocated to the different strata, and weights were used to ensure the representation of the survey results at the national level and the level of various geographical regions, with additional weights for the domestic violence subsample. The final sample included 34,282 households, with 20,481 eligible ever-married women of reproductive age (15–49 years), yielding a response rate of 93.4%.

The CGGI was developed for the 47 communities representing the 47 strata in the survey sample, which comprised the urban and rural segments of the 26 governorates included in the survey.

### GCCI for Egypt

#### GCCI Indicators

The index includes five indicators. Table 1 presents the questions underlying these five indicators, along with their coding scheme. No missing data were recorded for the indicated questions, with the final analytical sample size being 20,481 women for all questions, except for the subsample on domestic violence, for which the analytical sample consisted of 9,071 ever-married women.

**Table 1:** GCCI indicators and their relevant questions in EFHS 2021

Indicator	Questions	Coding
Child marriage	Now, I would like to ask you about your first husband. In what month and year did you start living together with your first husband?	1 = married before 18 years of age 0 = otherwise
No decision-making power	• Who decides how your husband’s earnings will be used? • Who usually makes decisions about health care for yourself: you, your husband, you and your husband jointly, or someone else? • Who usually makes decisions about making major household purchases? • Who usually makes decisions about visits to your family or relatives?	1 = not taking a decision on all 0 = otherwise
Wife-beating justifiable	In your opinion, is a husband justified in hitting or beating his wife in the following situations: a) If she goes out without telling him? b) If she neglects the children? c) If she argues with him? d) If she refuses to have sex with him? e) If she burns the food?	1 = justify wife-beating for any reason 0 = otherwise
Physical violence in the past 12 months	Did your (last) husband ever do any of the following things to you? • Push you, shake you, or throw something at you? • Slap you? • Twist your arm or pull your hair? • Punch you with his fist or with something that could hurt you? • Kick you, drag you, or beat you up? • Try to choke you or burn you on purpose? • Threaten or attack you with a knife, gun, or other weapon? • Physically force you to have sexual intercourse with him when you did not want to? • Physically force you to perform any other sexual acts you did not want to? • Force you with threats or in any other way to perform sexual acts you did not want to? How often did this happen during the last 12 months: often, only sometimes, or not at all? • Ever • Often • Sometimes • Not in the past 12 months	1 = exposure to any physical violence in the past 12 months 0 = otherwise
Less than secondary education	Have you ever attended school? • Yes • No What is the highest level of school you attended? • Primary • Preparatory • Secondary • Upper intermediate • University • More than university	1 = less than secondary 0 = secondary or more

#### GCCI Construction

The prevalence of the five indicators was calculated for the 47 different communities, resulting in a new dataset that was used to construct the index. Table 2 shows descriptive summary statistics of the indicators.

**Table 2:** Descriptive summary of the indicators at the level of the strata (*n* = 47)

Indicator	Mean	Std. Dev.	Min	Max
Child marriage	0.21	0.09	0.10	0.41
No decision-making power	0.22	0.11	0.10	0.72
Wife-beating justifiable	0.17	0.11	0.02	0.45
Physical violence in the past 12 months	0.11	0.06	0.00	0.30
Less than secondary education	0.36	0.17	0.11	0.96

Cronbach’s alpha coefficient was used to assess the internal consistency among the five indicators (Table 3). While excluding physical violence in the past 12 months increases internal consistency, a decision was made to retain it due to its significance in defining women’s status within their communities. This yielded a coefficient of 0.743, indicating an acceptable level of internal consistency.

**Table 3:** Cronbach’s alpha statistics for the five indicators at the level of the strata (*n* = 47)

Indicator	Sign	Item-test correlation	Item-rest correlation	Average interitem covariance	Alpha
Child marriage	+	0.85	0.77	0.0041	0.627
No decision-making power	+	0.69	0.48	0.005	0.706
Wife-beating justifiable	+	0.68	0.49	0.005	0.703
Physical violence in the past 12 months	+	0.34	0.21	0.007	0.776
Less than secondary education	+	0.89	0.71	0.003	0.623
Test scale				0.005	0.743

The one-factor exploratory factor analysis yielded one factor that accounted for 72.7% of the total variance, with all factor loadings exceeding 0.3. This shows a moderate correlation between the item and the factor [43–45]. Table 4 presents the factor loadings, uniqueness, and scoring of the rotated indicators. The resulting factor showed a mean of zero, a range between − 1.50 and 2.14, and a standard deviation of 0.938.

**Table 4:** Rotated factor loading, uniqueness, and scoring for the five indicators

Indicators	Factor loading	Uniqueness	Scoring
Child marriage	0.83	0.30	0.254
No decision-making power	0.57	0.68	0.098
Wife-beating justifiable	0.64	0.59	0.230
Physical violence in the past 12 months	0.33	0.89	0.130
Less than secondary education	0.85	0.28	0.496

K-means cluster analysis was used to categorize the GCCI into distinct homogeneous categories for descriptive analysis. Testing for the optimal number of clusters using the Calinski-Harabasz pseudo-F index, Table 5 shows that classifying the Gendered Cultural Context Score (GCCS) into four distinct clusters produced the highest Calinski-Harabasz pseudo-F score, indicating that this was the best partitioning for the score. According to the mean value of the measure, the four clusters were labeled least restrictive, less restrictive, restrictive, and most restrictive.

**Table 5:** The K-means cluster analysis for the GCCI

Number of clusters	Calinski/Harabasz pseudo-F Score	Number of communities in each cluster
3	130.04	11, 23, 13
4	201.61	11, 9, 17, 10
5	195.11	5, 14, 11, 8, 9

The four clusters of communities showed a gradual increase in the average prevalence of the five initial attributes (Table 6). While child marriage accounted for more than one-third of women in the most restrictive communities, slightly more than 13% were married before age 18 in the least restrictive communities. Similar patterns can be observed for the other four attributes.

**Table 6:** Indicators’ mean according to the four categories of the gendered cultural context score (GCCS)

Attributes	Least restrictive %	Less restrictive %	Restrictive %	Most restrictive %
Child marriage	13.3	17.0	22.5	34.9
No decision-making power	15.1	20.6	24.3	34.2
Wife-beating justifiable	6.3	13.8	22.1	26.4
Physical violence in the past 12 months	4.6	9.5	12.8	10.3
Less than secondary education	20.2	28.4	38.6	62.8

#### Gendered cultural context index and reproductive health

Table 7 presents the prevalence of some reproductive health risks according to the four GCCS clusters used to assess the validity of the GCCI in capturing disparities in these dimensions. It also presents the Wagstaff concentration index (CI) to assess the inequalities in these dimensions using the GCCI. Table 7 confirms the ability of the GCCI to capture disparities in reproductive health risks. Overall, the prevalence of these risks increases gradually with the move from the least restrictive communities to the most restrictive ones. This result was also confirmed by the positive value of the Wagstaff concentration index, indicating the more significant burden of these risks among those living in the most restrictive communities. The only exception to this positive sign was delivery by cesarean section, which was highly prevalent among the least restrictive communities. Additionally, except for only four risks, all other risks showed a concentration index magnitude of more than 0.10, indicating high levels of inequality by the GCCS.

**Table 7:** Proportions of reproductive health risks by the GCCS categories and Wagstaff concentration index

Reproductive health risks	Least restrictive %	Less restrictive %	Restrictive %	Most restrictive %	Wagstaff CI
Fertility-related					
- Multiparity (5 + child)	6.6	8.4	9.0	23.6	0.19
- Want more children	17.7	22.1	22.4	30.5	0.10
- Never use of contraceptives	10.6	9.9	9.4	14.0	0.06
- No current use of contraceptives	29.1	26.9	26.1	36.9	0.08
Pregnancy-related					
- No prenatal care	1.3	2.3	3.5	4.7	0.16
- No regular prenatal care	3.7	5.6	5.3	11.2	0.21
- Home delivery	1.0	2.3	3.2	10.6	0.35
- Delivery by cesarean section	84.6	79.9	78.5	70.6	−0.12
- No postnatal care	11.4	13.3	16.4	19.9	0.11
Experience pregnancy loss or child death					
- Miscarriage or pregnancy loss	23.7	26.6	24.8	33.4	0.08
- Child death	2.0	2.7	4.2	5.3	0.17
Other SRH issues					
- No knowledge of premarital examination	14.5	18.4	18.7	30.1	0.15
- No knowledge of STDs	39.7	43.2	45.4	66.6	0.20
- Have no decision power in seeking self-healthcare services	11.6	15.5	17.1	16.2	0.04
Consanguinity	16.3	24.8	27.9	47.0	0.24
Female Genital Mutilation (FGM					
- Having been subject to FGM	75.9	84.6	83.7	91.8	0.15
- Plans to subject her daughters to FGM	6.9	9.6	11.7	30.7	0.32
- Husbands prefer women subject to FGM	10.8	19.5	19.9	38.9	0.24
- FGM is required by religion	18.4	22.5	25.5	38.4	0.17
Access to information					
- Do not read the newspaper	73.0	76.0	75.7	89.4	0.18
- Do not own a mobile phone	5.2	12.6	16.7	25.3	0.27
- Do not own a computer	80.4	85.3	86.7	95.3	0.28
- No use of the internet	23.3	41.4	46.3	69.0	0.30
Financial independence					
- Not working	78.1	81.0	84.4	89.8	0.17
- Working for no pay or pay in kind only	2.7	5.7	6.3	7.2	0.16

By testing the GCCI’s ability to elucidate a portion of the observed variation at the community level, we have validated its role as a significant contextual factor influencing reproductive health. The results of the multilevel logistic regression models for multiparity, as detailed in Table 8, confirm this. The null model (Model 1) demonstrated that multiparity is indeed clustered at the community level, with a statistically significant level-2 variance of 0.428 (SE: 0.080) for multiparity. The intraclass correlation coefficient (ICC), or the percentage of variance attributable to level 2, was 11.4% of the total variance for multiparity. Model 2, which incorporated individual-level attributes, further supported the GCCI’s influence. In general, all individual attributes exhibited the expected effects on multiparity, with the odds of multiparity increasing with age, marrying at a young age (< 18), and experiencing child death, and decreasing with higher educational attainment and wealth. Overall, controlling for individual attributes increased the level-2 variance slightly to 0.465 (SE: 0.092) and increased the ICC to 12.4%. This result underscores the significant differences among communities, further highlighting the importance of the GCCI.

Model 3 introduces the GCCS, capturing 86.2% of the level 2 variance in multiparity. The ICC decreased from 12.4% to 6.7%, indicating the GCCS’s ability to explain a significant portion of the community variability in terms of multiparity. Model 3 also showed that adding the GCCS did not affect the coefficients of the individual-level attributes. For the GCCS, the odds of multiparity gradually and significantly decrease when moving from the most restrictive communities to the least restrictive ones. The odds of multiparity are slightly more than four times greater in the most restrictive communities than in the least restrictive ones. The likelihood ratio test between Model 2 and Model 3 was highly significant.

**Table 8:** Odds ratios for random intercept models assessing the associations of multiparity with individual- and community-level GCCS

	Model 1 Null model	Model 2 Individual attributes		Model 3 Individual attributes + GCCS	
**Individual-level attributes**		$\:\beta\:$	95% CI		95% CI
Age		1.14	(1.13 1.15)	1.14	(1.13 1.16)
Educational attainment (ref: less than secondary)					
Secondary		0.84	(0.73 0.97)	0.85	(0.73 0.98)
University and above		0.57	(0.42 0.78)	0.57	(0.42 0.78)
Wealth index (ref: poorest)					
Poor		0.81	(0.73 0.91)	0.82	(0.73 0.92)
Middle		0.62	(0.50 0.78)	0.64	(0.51 0.79)
Rich		0.50	(0.41 0.62)	0.52	(0.42 0.64)
Richest		0.41	(0.32 0.53)	0.43	(0.33 0.56)
Early married		2.81	(2.56 3.09)	2.80	(2.55 3.08)
Experienced child death		4.99	(3.80 6.55)	4.96	(3.78 6.52)
**Community level attribute**					
**GCCS**(ref: most restrictive)					
Restrictive				0.324	(0.22 0.56)
Less restrictive				0.356	(0.22 0.56)
Least restrictive				0.243	(0.14 0.40)
**Random effect**					
Level-2 variance^a^	0.423 (0.080)	0.472 (0.092)		0.240 (0.050)	
∆ (%) in level-2 variance		109.9%		51.0%	
ICC	11.4%	12.54%		6.8%	
−2 loglikelihood	15177.622	11836.56		11809.88	

a: values between brackets are the standard error for the level-2 variance

## Discussion

The current study proposes the construction of a contextual index for gender that reflects the cultural norms shaping women’s lives. These norms affect women’s health status and behaviors and contribute to their health disparities. In creating this index, we followed the criteria proposed in OECD/European Union [46] for constructing composite indicators. These criteria require conceptual relevance, no ambiguity, reliability, value-added, and power of discrimination. Addressing conceptual relevance, the initial step involved identifying the index variables. In developing the GCCI, our main concern is to identify women’s attributes that reflect a context in which women are undermined and suffer from limited opportunities to achieve their full potential. Another criterion used was the accessibility of the variables selected, which meets the OECD reliability criterion [46]. While many international gender indices require different data sources, we sought to work with variables that are usually available in regularly conducted large-scale datasets, such as the Demographic and Health Surveys, Family Health Surveys, or Multiple Indicator Cluster Surveys. This criterion enables the monitoring of comparisons within and across countries. For the study of reproductive health, the five selected attributes have repeatedly been reported to be strongly related to women’s status in communities. They are also closely linked to reproductive health. Attaining less than a secondary education has been consistently linked to hindering social change [30]. Furthermore, early marriage, justification of and exposure to violence, and lack of decision-making power in the family are associated with the low status of women and the denial of their fundamental human rights. The widespread overlap and interdependence of these attributes can create a restrictive context for women, limiting their life opportunities. The GCCI is a summary index of these attributes at the community level. It responds to the no-ambiguity criterion in the OECD methodology by allowing the classification of the communities on a continuum that ranges from the most restrictive, where high prevalences of these attributes are observed, to the least restrictive, where the prevalence of these attributes is low.

The validity of the proposed index was tested using data from the 2021 Egyptian Family Health Survey, and the findings revealed its ability to discriminate and differentiate among communities. These findings have significant implications for understanding and addressing reproductive health disparities. A comparison across the four categories of the GCCS revealed that a move from the most restrictive communities to the least restrictive ones is associated with a higher likelihood of having lower fertility with no desire for more children, which translates into the ever and current use of contraceptives. The utilization of maternal care services during pregnancy and delivery also increased with this move. This use of services was reflected in their low chances of experiencing pregnancy loss or child death. This same move is also associated with a higher likelihood of having more knowledge of other reproductive health risks and services, including sexually transmitted diseases and premarital examinations, and an increase in the exercise of decision-making power over seeking healthcare for themselves. As expected, traditional attitudes around consanguinity and FGM-related behaviors and attitudes were highly prevalent in more restrictive communities.

The findings also highlighted the fact that communication channels for sharing information with women living in restrictive communities are relatively limited, which calls for urgent and innovative approaches in health promotion campaigns. Furthermore, women’s economic independence through work and money-earning power is very limited in these communities, increasing their reliance on family and diving deeper into the prevailing cultural norms. The Wagstaff concentration index confirmed all the above findings, with positive values and magnitudes exceeding the 0.10 threshold. Furthermore, the multilevel analysis revealed that, after controlling for individual-level attributes, the GCCS accounts for 86.2% of the level-2 variance in multiparity. The ICC decreased from 12.4% to 6.7%, indicating that the GCCS’s ability to explain almost 50% of the community variability in terms of multiparity did not affect the coefficients of the individual-level attributes. Furthermore, the odds of multiparity gradually and significantly decrease as one moves from the most restrictive communities to the least restrictive ones, with the odds of multiparity being approximately four times greater in the most restrictive communities than in the least restrictive ones.

These findings support the shift away from viewing gender as merely an individual-level attribute, which often highlights differences between males and females, with other vulnerabilities assessed through the intersectionality of gender with other individual attributes [47–49] or the use of indices, such as reproductive health empowerment measures, which, while focusing on women only, capture the complexity of the relationship between various individual attributes and women’s reproductive health [50].

These findings also support the expansion of previous research that examined the impact of the context in which women live, with its limited focus on the physical features and service availability on their health [51, 52], to incorporate the gendered cultural context. By capturing this context, the GCCI provides a unique and comprehensive tool for understanding and addressing the impact of the cultural norms on reproductive health disparities across various communities. The comprehensive nature of the GCCI ensures that no aspect of the gendered cultural context is overlooked, providing a thorough understanding of the issue.

However, these results also highlight some relevant limitations in this approach. The aim of the proposed approach is to define the gendered cultural context for small communities within a specific country and to classify these communities on a continuum from the most restrictive to the least restrictive. Since different countries have varying gendered cultural norms, using the same indicators to construct the index across different countries does not allow for accurate cross-country comparisons. However, employing the same approach to classify communities from most to least restrictive allows for comparison based on this classification. Furthermore, in applying the CGGI approach to Egypt, we selected indicators identified in the international literature. A more refined application of the approach in different cultural contexts would require extensive study and conceptualization of the influential gendered cultural norms prevailing in the countries under investigation. This would include ensuring that large-scale surveys incorporate some indicators that reflect and operationalize these identified norms. Additionally, as a potential approach to studying the gendered cultural context of individuals’ health and well-being, the conceptualization process should be expanded to address the specific health and well-being dimension and the target population. In other words, the gendered cultural context around older women suffering from Alzheimer’s calls for a different conceptualization than that affecting men suffering from hypertension. These limitations underscore the need for further research and refinement of the approach.

## Conclusion

Despite recognizing the impact of gender norms on women’s well-being, limited efforts have been made thus far to assess the effects of these norms at the level of small communities. The Gendered Cultural Context Index offers an opportunity to utilize data from large-scale surveys to develop a community-level index that measures reproductive health inequality across diverse communities. While we attempted to use attributes regularly reported in these surveys in the current study, the approach implemented in developing the index can be adapted to utilize different country-specific cultural factors that affect individual well-being. This study opens the door for future research to further refine and validate the GCCI, and to explore its potential applications in other cultural contexts and for other health and well-being dimensions.

In conclusion, due to its simple structure and data availability, the proposed index and its development approach provide a solid foundation for further efforts to capture the contextual social and cultural norms that shape women’s health. These efforts can facilitate the development of policy recommendations that consider their cumulative impact on women’s reproductive and overall health, leading to the adoption of more effective social policies and interventions.

## Data Availability

The dataset analyzed during the current study is available at the Egyptian Central Agency for Public Mobilization and Statistics (CAPMAS) repository: https://censusinfo.capmas.gov.eg/Metadata-en-v4.2/index.php/catalog/665.
